# The Longitudinal Dyadic Associations Between Social Participation and Cognitive Function in Older Chinese Couples

**DOI:** 10.1093/geronb/gbae045

**Published:** 2024-04-12

**Authors:** Jianhua Hou, Tianyong Chen, Nancy Xiaonan Yu

**Affiliations:** Department of Social and Behavioural Sciences, City University of Hong Kong, Kowloon, Hong Kong, People’s Republic of China; Institute of Psychology, Chinese Academy of Sciences, Beijing, People’s Republic of China; Department of Psychology, University of Chinese Academy of Sciences, Beijing, People’s Republic of China; Department of Social and Behavioural Sciences, City University of Hong Kong, Kowloon, Hong Kong, People’s Republic of China; (Psychological Sciences Section)

**Keywords:** Cognitive function, Dyadic perspective, Health concordance, Lagged dependent actor–partner interdependence model, Social participation

## Abstract

**Objectives:**

Based on the “linked lives” tenant of the life course perspective, this longitudinal study aims to examine the actor and partner effects of social participation on cognitive function in older Chinese couples.

**Methods:**

A total of 1,706 couples aged 60 and older were included in the final analyses. Social participation was measured using 2 questions regarding types of activities and frequency. Cognitive function was measured using a combination of memory, orientation, visuoconstruction, attention, and calculation. The lagged-dependent APIM was used to model the dyadic associations between social participation and cognitive function.

**Results:**

The time-averaged actor effects of both husbands’ and wives’ social participation on their own cognitive function were significant (*p* < .001 for both). The time-averaged partner effect of husbands’ social participation on wives’ cognitive function was significant (*p* < .001) but the reverse—the effect of wives’ social participation on husbands’ cognitive function—was not (*p* = .381). The time-specific actor and partner effects were not significant (*p* > .05 for all).

**Discussion:**

Our findings indicate an asymmetrical pattern of actor–partner interdependence, where husbands’ social participation may affect their wives’ cognitive function on average, but wives’ social participation does not affect their husbands’ cognitive function. Clinical practitioners should invite both partners, especially husbands, to participate in social participation interventions to facilitate crossover benefits for wives. Moreover, policymakers should build more facilities to encourage older couples to engage in social activities to prevent cognitive decline.

According to the Seventh National Census issued by the National Bureau of Statistics of China, China has the highest population of older individuals in the world, with 264 million aged 60 and older accounting for 18.7% of the total population. Cognitive impairment is one of the most common mental disorders among older adults, with an estimated prevalence of 20.4% (Yuan et al., 2021). Cognitive impairment not only hinders daily functioning (Parikh et al., 2015) but also undermines the quality of life (Hussenoeder et al., 2020). In terms of healthcare economics, the annual cost related to cognitive impairment will rise from $248.71 billion in 2020 to $1.89 trillion in 2050 in China (Jia et al., 2021), which may constitute huge public and socioeconomic burdens. Therefore, delaying or preventing cognitive decline is a critical public health issue in an aging society.

## Social Participation and its Relationship With Cognitive Function

Participation in social activities refers to an individual’s involvement in activities providing social interactions with others based on personal interests in social life and important shared spaces (Levasseur et al., 2022). Social participation is a crucial element in creating age-friendly communities that encourage active aging as people grow older (WHO, 2007). There is a wealth of research linking social participation with better cognitive function (Sakamoto et al., 2017), slower cognitive decline (Samtani et al., 2022), and reduced odds of developing dementia (Wang & Xia, 2020). This phenomenon may be explained by the concept of cognitive reserve (Stern, 2002), which suggests that the brain can compensate for age-related changes by utilizing pre-existing cognitive processing strategies or engaging compensatory mechanisms (Stern et al., 2020). Engaging in cognitive-stimulating activities like education, occupation, and leisure pursuits can increase cognitive reserve, which may help to mitigate the effects of aging on cognitive function by recruiting alternative brain regions or operating brain networks more efficiently (Steffener et al., 2011). Furthermore, according to the mental exercise hypothesis (Salthouse, 2006), engaging in mentally stimulating activities can help maintain cognitive function and even reverse the effects of cognitive decline. While past experience (i.e., education levels and occupation) may be difficult or impossible to modify for older adults, social participation plays a unique role in shaping cognitive reserve during older adulthood (Grotz et al., 2017).

## The Importance of Couple Relationship

The reciprocal effects of couples’ social participation in older adulthood are of significant interest for several theoretical and practical reasons. First, the life course principle of “linked lives” posits that individual behaviors and outcomes are often influenced by those of proximate others, particularly those with whom one may share a close relationship (Elder & O’Rand, 1995). Marriage is a critical means by which individuals’ lives are linked together. Through the sharing of resources and social control mechanisms that promote healthy behavior, married individuals tend to experience better overall health and cognitive function than unmarried people (Hakansson et al., 2009; Monserud, 2019). These associations remain even after researchers control for selection effects, such as the tendency for healthy individuals to be more likely to enter into marriage. Spousal relationships are often intimate in nature, strengthening the reciprocal effects of partners on one another via the provision of social and emotional resources (Hoppmann & Gerstorf, 2009). For older adults, spousal relationships are of increasing importance since social networks tend to shrink to those that are the most emotionally and socially rewarding (Zheng & Chen, 2020).

Hoppmann and Gerstorf (2014) also offers a working model to explain the link between health behaviors and health outcomes in older couples. There are two possible ways a partner’s social participation can influence the other partner’s cognitive function: (a) shared goals. When older couples have common or interrelated goals (e.g., staying socially active to age well together), then one partner’s social participation automatically affects the other partner’s social participation (since they achieve their goal as a team), which, in turn, may influence both partners’ cognitive functioning; (b) shared problem-solving. When one partner’s social participation declines due to severe illness or dysfunction, the other partner may compensate for it at the cost of their own social participation, which may influence both partners’ cognitive functioning. Thus, these mechanisms may drive the actor and partner effects of social participation on cognitive functioning in older couples.

Second, empirical evidence has shown that levels of social participation are highly correlated within older couples (Ertel et al., 2008; Weber & Hulur, 2021). Moreover, concordance within couples exists regarding cognitive function (Dufouil et al., 2000; Yang et al., 2021), social participation (Hoppmann et al., 2008), and mental and physical condition (Caillot-Ranjeva et al., 2022; Kang et al., 2020). However, only a few studies have directly assessed the association between one spouse’s social participation and the cognitive function of the other spouse.

A recent study conducted in both the United States and Mexico has directly examined the association between social participation and cognitive function within couples, revealing that in Mexico, wives’ social participation had a positive effect on their husbands’ cognitive function (*β* = 0.08, *p* < .01), but husbands’ social participation did not impact their wives’ cognitive function. However, in the United States, no partner effects were observed at all (Howrey et al., 2021). These findings reflect the cultural differences between both countries since the United States prioritizes individualism although Mexico tends to prioritize codependence and collectivism (Hofstede, 2001). In another study conducted in Australia, husbands’ social participation was associated with their wives’ perceptual speed, but there was no correlation between wives’ social participation and their husbands’ perceptual speed (Hoppmann et al., 2008). More broadly, substantial individual-level studies consistently documented the gender differences in the benefits of social participation, with some studies suggesting that women derive greater benefits from social participation (Thomas, 2011), whereas others indicate that men benefit more (Aida et al., 2011; Pothisiri & Vicerra, 2021).

The health concordance of older married couples has mainly been studied with data from Western countries such as the United States, where certain gender norms may influence the health outcomes of men and women. However, the existence and extent of such concordance in countries with different gender norms remains unclear. The Chinese government deems marriage as a crucial institution for political stability, whereas Chinese society views it as a symbol of social normalcy and responsibility to one’s family (Wang & Xia, 2020). Confucianism and collectivism have significantly influenced Chinese families by emphasizing male dominance in interdependence (e.g., “The husband sings, and the wife follows,” known as “夫唱妇随”) and relational harmony (e.g., “The lute and psaltery are in harmony,” or “琴瑟和鸣”; Park & Chesla, 2007). It is currently unclear how the traditional gendered roles of husbands and wives in China affect the influence of social participation on cognitive function and whether findings from previous studies can be generalized to other cultural backgrounds like China.

## Actor–Partner Interdependence Model and its Longitudinal Extension

The Actor−Partner Interdependence Model (APIM) is widely used in the analysis of dyadic data to account for the nonindependence of two dyad members (Cook & Kenny, 2005). The APIM tests how one dyad member’s behaviors predict the other dyad member’s outcomes (partner effects), above and beyond the effects of each dyad member’s behaviors on his or her own outcomes (actor effects). The longitudinal extension of the APIM (ie, the Lagged Dependent Actor–Partner Interdependence Model, LDAPIM) allows for both stable and time-varying sources of nonindependence (Gistelinck & Loeys, 2019). First, this model can account for the nonindependence between the two partners (i.e., covarying dependent variables). For instance, the cognitive function of a specific wave between two dyad members will show more similarities compared to two random people. Ignoring this nonindependence will lead to biased estimates as it violates the regression assumption of independent observations (Kenny, 1995). Second, this model can account for the nonindependence of the repeated observations within a dyad member (i.e., lagged effect). This type of nonindependence depicts the association between a variable measured at one time point and the same variable measured at another time point. For example, cognitive function at one time point can be positively associated with the cognitive function at another time point for two dyad members. Ignoring this temporal correlation may also lead to biased estimates (Hox et al., 2010). Third, this model can simultaneously assess the effect of each dyad member’s behaviors on their own outcomes (the actor effect), and the effect of one dyad member’s behavior on the other dyad member’s outcomes (the partner effect). For example, one dyad member’s social participation may affect not only their own cognitive function but also their partner’s. Fourth, this model can separate the average effect over time (the time-averaged effect) and the deviant effect towards this average at a particular wave (the time-specific effect; Gistelinck & Loeys, 2019). For example, the average level of social participation over time may have a different effect between persons on the cognitive function than a sudden drop or increase in social participation within a person on a wave. If ignored, the estimated effect will be a mixture of time-averaged and time-specific effects (Enders & Tofighi, 2007).

## Research Questions and Hypotheses

Guided by the “linked lives” tenant of life course perspective, this study aims to assess the following three research questions: First, we examine how social activities are related to cognitive function in couples. We hypothesized that (a) a spouse’ cognitive function relates not only to their own social activities (H1, the time-averaged actor effect) but also to those of the other spouse on average (H2, the time-averaged partner effect) and (b) a spouse’s cognitive function related not only to their own increase or decrease in social activities at a specific wave (H3, the time-specific actor effect) but also to that of the other spouse (H4, the time-specific actor effect).

Second, we investigated the lagged and cross-lagged relationship among cognitive function across four waves. We hypothesized that (c) a spouse’s cognitive function at one wave relates not only to their own cognitive function at the previous wave (H5, lagged effect) but also to those of the other spouse (H6, cross-lagged effect).

Third, we examined the interdependence of cognitive function between two individuals in a dyad in terms of their average cognitive function over time and at specific waves. We hypothesized that there is a significant correlation between two dyad members’ average cognitive function (H7, time-averaged covary) as well as a significant correlation between two dyad members at a particular wave (H8, time-specific covary).

## Method

This study was reported according to the Strengthening the Reporting of Observational Studies in Epidemiology (STROBE) Checklist (see Supplementary Table 1; von Elm et al., 2007).

### Participants

The analyzed data were extracted from the four-wave (2011, 2013, 2015, and 2018) Chinese Health and Retirement Longitudinal Study (CHARLS) harmonized dataset, which is a survey of middle-aged and older adults in 450 villages or urban communities, 150 counties or districts, and 28 provinces in China (Zhao et al., 2014). The CHARLS sample is representative of people aged 45 and older living in households. The sampling method was a four-stage process: country-level, neighborhood-level, household-level, and respondent-level. The detailed sampling method can be found at https://charls.charlsdata.com/index/en.html. All participants provided written informed consent, and survey protocols were approved by the Peking University Ethics Review Board (Zhao et al., 2013). To be included in the current study, participants needed to (a) be older than 60 years old at baseline; (b) be in a marital relationship with another participant; (c) have completed at least one wave of the assessment of social participation and cognitive function. Missing data were estimated using the full information maximum likelihood (FIML). The screening procedure is shown in Supplementary Figure 1. A total of 1,706 couples were included in the final analyses.

### Measures

We treated baseline demographic information, including age (“60–69,” “70–79,” and “80 and above”), education level (0 = “none,” 1 = “less than lower secondary,” 2 = “upper secondary and vocational training,” 3 = “tertiary”), and Hukou (0 = “urban,” 1 = “rural”) as time-invariant control variables and self-reported health status and depression as time-varying control variables. Self-reported health status was measured using a single-item scale with five points (“Would you say your health is excellent, very good, good, fair, or poor?”) in each of the four waves. Depression was measured using the Center for Epidemiology Studies of Depression (CESD-10) scale with a cutoff score of 10 (Cheng & Chan, 2005). This scale has been validated and widely used for older Chinese adults (Cheng & Chan, 2005).

Social participation was measured using two multiple-choice questions: “Have you done any of these activities in the last month?” and “How often in the last month do you attend that activity?” For the first question, there were 10 options, seven of which were considered social participation activities, including (a) interacting with friends, (b) playing mahjong, chess, or cards or going to a community club, (c) going to a sport, social, or other kind of club, (d) taking part in a community-related organization, (e) volunteering, (f) caring for a sick or disabled adult who does not live with you and who did not pay you for the help, and (g) attending an educational or training course. Following a previous publication using the same dataset, the remaining three options, “providing help to family, friends, or neighbors who do not live with the respondent and who do not pay for the help,” “investing in stocks,” and “surfing the internet” were not considered typical social participation activities in the Chinese setting (Hu et al., 2012). For the second question, there were three options, including “almost daily,” “almost every week,” and “not regularly.” Considering that the raw scores of the respondents were highly right-skewed, we combined the responses to the two questions and categorized their responses into three groups: (2 = attending any one of the social activities with a frequency of almost daily, 1 = attending any one of the social activities with a frequency of almost every week or not regularly, and 0 = not attending any one of the social activities). The distribution of each response was shown in Supplementary Table 2.

Cognitive function was measured by a set of four tests that assessed (a) memory by an immediate recall and a delayed recall of 10 Chinese words; (b) orientation by requiring participants to answer the date (i.e., month, day, and year); (c) visuoconstruction by asking participants to copy a figure of two overlapping pentagons; and (d) attention and calculation ability by asking participants to subtract 7 from 100 (up to five times). All cognitive measures were from the Telephone Interview of Cognitive Status (TICS), a well-established scale to measure individual cognitive status and monitor changes in cognitive functioning over time (Cook et al., 2009). We calculated a total score ranging from 0 to 30 for these tests to represent general cognitive function, with higher scores indicating better cognitive function. The average scores on the cognitive measure from Wave 1 to Wave 4 were 14.67 (*SD* = 4.39), 14.75 (*SD* = 4.50), 13.53 (*SD* = 4.53), and 14.40 (*SD* = 5.00) for husbands, and 13.45 (*SD* = 4.93), 13.47 (*SD* = 4.93), 12.42 (*SD* = 5.24), and 13.31 (*SD* = 5.67) for wives. The cognitive function measure had moderate internal consistency and good test–retest reliability (*α*_w1_ = 0.78, *α*_w2_ = 0.78, *α*_w3_ = 0.79, *α*_w4_ = 0.77, ICC = 0.78 for husbands and *α*_w1_ = 0.78, *α*_w2_ = 0.78, *α*_w3_ = 0.80, *α*_w4_ = 0.77, ICC = 0.77 for wives).

### Statistical Analysis

Descriptive statistics were calculated for all included variables, and Spearman’s correlations between social participation and cognitive function in each of the four waves were analyzed. Next, we followed the established method to construct an LDAPIM (Gistelinck & Loeys, 2019). We proposed the following model equations for the current study:


$$\begin{array}{l} \left\{ \begin{array}{lllllll} & CF_{hij}=\mu_{h}+\rho_{h}CF_{h,i-1,j}+\lambda_{w}CF_{w,i-1,j}+a_{h(SPA)}SPA_{hj}\; & \;\;\;\;\;\;\;\;\;\;\;\;\;+p_{wh(SPA)}SPA_{wj}+a_{h(SPS)}SPS_{hij}+p_{wh(SPS)}SPS_{wij}+\eta_{hj}\; & \;\;\;\;\;\;\;\;\;\;\;\;\;+\varepsilon_{hij}\; & CF_{wij}=\mu_{w}+\rho_{w}CF_{w,i-1,j}+\lambda_{h}CF_{h,i-1,j}+a_{w(SPA)}SPA_{wj}\; & \;\;\;\;\;\;\;\;\;\;\;\;\;+p_{hw(SPA)}SPA_{hj}+a_{w(SPS)}SPS_{wij}+p_{hw(SPS)}SPS_{hij}+\eta_{wj}\; & \;\;\;\;\;\;\;\;\;\;\;\;\;+\varepsilon_{wij}\; \end{array}\right. \end{array}$$

with *i* referring to the time point (1–4) and *j* to the dyad number (1–1,706). *CF* represents cognitive function; SP corresponds to social participation. Moreover, $\rho_{h}$ and $\rho_{w}$ refer to autoregression estimate, $\lambda_{h}$ and $\lambda_{w}$ refer to the cross-lagged association, $a_{h\left(SPA\right)}$ and $a_{w\left(SPA\right)}$ refer to the time-averaged actor effect, $p_{wh\left(SPA\right)}$ and $p_{hw\left(SPA\right)}$ refer to the time-averaged partner effect, $a_{h\left(SPS\right)}$ and $a_{w\left(SPS\right)}$ refer to the time-specific actor effect, and $p_{wh\left(SPS\right)}$ and $p_{hw\left(SPS\right)}$ refer to the time-specific partner effect.

The following represents the residual covariance structure at a particular wave, with *j* corresponding to the dyad identification number:


$$\begin{array}{l} Cov\left(\begin{array}{l} \varepsilon_{hj}\;\varepsilon_{wj}\; \end{array}\right)=\left(\begin{array}{lll} \sigma_{Yh}^{2} & \sigma_{Yhw}\;\sigma_{Yhw} & \sigma_{Yw}^{2}\; \end{array}\right) \end{array}$$

For the random intercept variances, we used an unstructured covariance structure:

$Cov\left(\begin{array}{l} \eta_{hj}\;\eta_{wj}\; \end{array}\right)=\left(\begin{array}{lll} \tau_{h}^{2} & \tau_{hw}\;\tau_{hw} & \tau_{w}^{2}\; \end{array}\right).$ The descriptive statistics were generated using SPSS 20.0 and LDAPIM was modeled by a user-friendly, free application, called the LDDinSEM-application (https://fgisteli.shinyapps.io/Shiny_LDD2/). The sensitivity analyses were used to check the robustness of our findings by comparing the statistical results from (a) the model controlling for demographic characteristics, self-rated health, and depression versus without these covariates and (b) the dataset with missing data versus the dataset without death-caused missing.

## Results

### Sample Characteristics and Bivariate Correlations

Among the 1,706 couples, wives tended to be younger, had a lower education level, held rural Hukou, had worse self-reported health status, and reported more depressive symptoms than husbands (*p* < .01; Table 1). Social participation and cognitive function remained stable over the four waves, though husbands’ cognitive function was slightly higher than that of their wives. Nearly half of the participants participated in at least one social activity (Supplementary Figure 2) and more than 20% of the participants chose “interacting with friends” and “playing mahjong, chess, cards, or going to a community club” (Supplementary Figure 3).

**Table 1.** Baseline Sample Characteristics (*N* = 1,706 couples)

Characteristic	Husband *n* (%)	Wife *n* (%)	χ^2^	*p*
Age			92.88	<.001
60–69	1,095 (64.2)	1,346 (78.9)		
70–79	551 (32.3)	335 (19.6)		
80 or above	60 (3.5)	25 (1.5)		
Education level			9.13	.01
No formal education	1,480 (86.8)	1,533 (89.9)		
Primary or below	149 (8.7)	119 (7.0)		
Above primary	77 (4.5)	52 (3.1)		
Hukou^a^			30.39	<.001
Urban	555 (32.5)	410 (24.0)		
Rural	1,149 (67.4)	1,294 (75.8)		
Self-reported health			31.54	<.001
Excellent	94 (5.5)	67 (3.9)		
Very good	270 (15.9)	233 (13.7)		
Good	875 (51.4)	799 (46.9)		
Fair	395 (23.2)	490 (28.8)		
Poor	69 (4.1)	113 (6.6)		
Depression			3.05	<.001
Yes	399 (23.4)	543 (31.8)		
No	1,089 (63.8)	1,229 (72.0)		

*Note*:

^a^Hukou is a system of household registration, which originated from ancient China over a 1,000 years. Hukou has implications in the inequality in education resources, healthcare support, retirement pension, etc. in China.

Table 2 displays the Spearman correlations between social participation and cognitive function. Concerning social participation, the correlations within individuals and between individuals across four waves were statistically significant, with correlation coefficients ranging from 0.08 to 0.39 (highlighted in yellow). However, there was one exception, which was the relationship between husbands’ social participation at the fourth wave and wives’ social participation in the second wave, where the correlation coefficient was 0.05. In terms of cognitive function, the correlations within individuals and between individuals across four waves were also statistically significant, with correlation coefficients ranging from 0.19 to 0.61. Additionally, social participation was found to have a positive association with cognitive function in most cases, with correlation coefficients ranging from 0.02 to 0.21.

**Table 2.** Spearman Correlations Between Social Participation and Cognitive Function

Variable	SP_H1_	SP_H2_	SP_H3_	SP_H4_	SP_W1_	SP_W2_	SP_W3_	SP_W4_	CF_H1_	CF_H2_	CF_H3_	CF_H4_	CF_W1_	CF_W2_	CF_W3_	CF_W4_
SP_H1_	—															
SP_H2_	**0.32**	—														
SP_H3_	**.27**	**.28**	—													
SP_H4_	**0.24**	**0.20**	**0.29**	—												
SP_W1_	**0.39**	**0.12**	**0.08**	**0.08**	—											
SP_W2_	**0.11**	**0.29**	**0.11**	0.05	**0.29**	—										
SP_W3_	**0.12**	**0.13**	**0.24**	**0.08**	**0.28**	**0.28**	—									
SP_W4_	**0.11**	**0.07**	**0.09**	**0.22**	**0.20**	**0.19**	**0.28**	—								
CF_H1_	**0.14**	**0.12**	**0.09**	**0.08**	**0.06**	0.01	0.03	0.00	—							
CF_H2_	**0.14**	**0.12**	**0.15**	**0.10**	**0.09**	**0.06**	**0.09**	0.05	**0.49**	—						
CF_H3_	**0.13**	**0.14**	**0.21**	**0.11**	0.05	0.05	**0.06**	0.04	**0.51**	**0.53**	—					
CF_H4_	**0.07**	0.02	**0.07**	0.04	0.04	0.03	**0.07**	0.06	**0.40**	**0.42**	**0.50**	—				
CF_W1_	**0.16**	**0.14**	**0.08**	**0.12**	**0.13**	**0.07**	**0.12**	**0.06**	**0.40**	**0.23**	**0.28**	**0.20**	—			
CF_W2_	**0.14**	**0.12**	**0.14**	**0.11**	**0.13**	**0.09**	**0.16**	**0.09**	**0.30**	**0.34**	**0.29**	**0.19**	**0.54**	—		
CF_W3_	**0.11**	**0.12**	**0.10**	**0.10**	**0.10**	**0.14**	**0.14**	**0.13**	**0.26**	**0.23**	**0.29**	**0.28**	**0.55**	**0.56**	—	
CF_W4_	**0.08**	0.05	0.06	0.08	**0.08**	**0.08**	**0.11**	0.06	**0.28**	**0.23**	**0.21**	**0.21**	**0.53**	**0.56**	**0.61**	—

*Notes*: CF = cognitive function; H = husband; SP = social participation; w = wife; 1–4 = 1st wave to 4th wave. Significant correlations are in bold.

### Time-Averaged Actor and Partner Effects

Table 3 lists the time-averaged actor and partner effects of social participation. The time-averaged effect of husbands’ social participation on their cognitive function was positive and significant (*b* = 1.07, *SE* = 0.18, *p* < .001, H1_husband_ confirmed), as was the time-averaged effect of wives’ social participation on their own cognitive function (*b* = 0.96, *SE* = 0.21, *p* < .001; H1_wife_ confirmed). This indicates that husbands and wives who irregularly participated in social activities had cognitive scores 1.07 and 0.96 points higher, respectively, than those who did not participate. However, those who engaged in almost daily social activities exhibited an even higher cognitive performance.

The time-averaged partner effect of husbands’ social participation on their wives’ cognitive function was positive and significant (*b* = 0.84, *SE* = 0.21, *p* < .001, H2_wife_ confirmed). However, the time-averaged partner effect of wives’ social participation on their husbands’ cognitive function was not significant (*b* = 0.15, *SE* = 0.17, *p* = .381; H2_husband_ not confirmed). This indicates that wives whose husbands irregularly participated in social activities had cognitive scores that were 0.84 points higher than those whose husbands did not participate in any social activities. However, wives whose husbands participated in social activities almost daily exhibited even better cognitive functioning.

### Time-Specific Actor and Partner Effects

Table 3 shows the time-specific actor and partner effects of social participation. The time-specific effect of husbands’ social participation on their cognitive functioning was not significant (*b* = 0.19, *SE* = 0.12, *p* = .099, H3_husband_ not confirmed), nor was the time-specific effect of wives’ social participation on their cognitive functioning significant (*b* = 0.05, *SE* = 0.13, *p* = .717, H3_wife_ not confirmed). For both husbands and wives, a rise in social activities at a specific wave (e.g., transitioning from never participating in social activities to participating irregularly) was not significantly correlated with improved cognitive performance.

The time-specific effect of the husbands’ social participation on their wives’ cognitive function was not significant (*b* = 0.19, *SE* = 0.14, *p* = .165, H4_wife_ not confirmed). Similarly, the time-specific effect of wives’ social participation on their husbands’ cognitive function was not significant (*b* = − 0.04, *SE* = 0.12, *p* = .743, H4_husband_ not confirmed). For both husbands and wives, an increase in social activities at a specific wave (e.g., transitioning from never participating in social activities to participating irregularly) did not exhibit a significant correlation with an enhancement in the cognitive performance of their partners.

### Lagged and Cross-lagged Effects

The autoregression parameters for husbands and wives were 0.16 (*SE* = 0.04, *p* < .001, Table 3) and 0.17 (*SE* = 0.04, *p* < .001), respectively (H5_husband_ and H5_wife_ confirmed). Thus, if husbands or wives had a higher level of cognitive function at a particular wave, their cognitive function would also be higher at the subsequent wave. The parameters for cross-lagged association were 0.10 (*SE* = 0.04, *p* < .001) for husbands and 0.01 (*SE* = 0.04, *p* = .85) for wives, respectively (H6_husband_ confirmed and H6_wife_ not confirmed). If wives showed a higher level of cognitive functioning at one wave, their husbands would exhibit an even higher level at the subsequent wave. However, the inverse was not significant.

### Time-Average Covary and Time-Specific Covary

lists the covariance parameter estimates. The upper part of the table contains the random effects covariance parameters, whereas the lower part contains the residual covariance parameters. Variation in average cognitive functioning was 6.81 and 10.81 for husbands and wives, respectively. The correlation of the average cognitive function between husbands and wives was $\frac{2.89}{\sqrt{6.81*10.81}}=0.34$ (H7 confirmed). The residual covariance of cognitive function on a particular wave was similar for husbands and wives: 11.41 for husbands as opposed to 12.42 for wives. The correlation of wave fluctuations between husbands and wives had a magnitude of $\frac{2}{\sqrt{11.41*12.42}}=0.17$ (H8 confirmed). Hence, when one dyad member experienced high cognitive functioning on a particular wave, so did the other.

**Table 3.** Parameter Estimates of the Fixed Effects (*N* = 1,706 couples)

Variable	*b*	*SE*	*p*	*Lower CI*	*Upper CI*
Husbands’ CF					
Intercept	**9.26**	0.60	<.001	8.07	10.44
Lagged	**0.16**	0.04	<.001	0.09	0.24
Cross-lagged	**0.10**	0.04	.004	0.03	0.17
Husbands’ SP_TA_	**1.07**	0.18	<.001	0.72	1.43
Wives’ SP_TA_	0.15	0.17	.381	−0.19	0.49
Husbands’ SP_TS_	0.19	0.12	.099	−0.04	0.42
Wives’ SP_TS_	−0.04	0.12	.743	−0.27	0.19
Wives’ CF					
Intercept	**8.64**	0.67	<.001	7.33	9.95
Lagged	**0.17**	0.04	<.001	0.09	0.25
Cross-lagged	0.01	0.04	.85	−0.07	0.08
Wives’ SP_TA_	**0.96**	0.21	<.001	0.55	1.36
Husbands’ SP_TA_	**0.84**	0.21	<.001	0.43	1.26
Wives’ SP_TS_	0.05	0.13	.717	−0.20	0.29
Husbands’ SP_TS_	0.19	0.14	.165	−0.08	0.46

*Notes*: CF = cognitive function; *CI* = confidence interval; *SE* = standard error; SP = social participation; TA = time-averaged effect; TS = time-specific effect. Significant correlations are in bold.

**Table 4.** Parameter Estimates of the Covariance Structure (*N* = 1,706 couples)

Variable	φ	*SE*	*p*	*Lower CI*	*Upper CI*
Between dyad covariance					
	6.81	1.04	<.001	4.77	8.85
	10.81	1.56	<.001	7.76	13.85
	2.89	1.00	.004	0.94	4.85
Within dyad covariance					
	11.41	0.50	<.001	10.44	12.38
	12.42	0.58	<.001	11.28	13.55
	2.00	0.44	<.001	1.14	2.86

*Notes*: *CI* = confidence interval; h = Husband; *SE* = standard error; w = wife.

### Sensitivity Analyses

Supplementary Tables 3–6 summarize the findings from sensitivity analyses. After controlling for demographics (i.e., age, education, and Hukou), self-reported health status, and depression, our findings remained unchanged. After deleting the death-caused missing data (*n* = 66 dyads), our findings were similar.

## Discussion

This is the first study examining the dyadic relationship between social participation and cognitive function among older Chinese couples. Our study provides longitudinal evidence, with four waves spanning 8 years. We examined the concordance of cognition within the couples as well as the dyadic influence of social participation on cognitive function in older couples.

First, both partners’ social participation positively predicted their own cognitive function on average (H1, time-averaged actor effect). However, an increase in a partner’s social participation did not predict their own cognitive function on a particular wave (H3, time-specific actor effect), which suggests that social participation may predict cognition over time without obvious fluctuations (Ding et al., 2022). Overall, social participation plays a protective role in cognitive function in older adults regardless of their demographics or health status. Our participants predominantly engaged in activities such as interacting with friends, playing cognitively stimulating games (e.g., Mahjong), and participating in sports or social clubs, suggesting that the positive effects of social participation may be largely attributed to these activities. The following are possible interpretations for these effects: (a) Participating in socially-focused activities (such as chatting with friends) may help older adults find meaning in life, maintain social connections and positive attitudes towards aging, feel a sense of usefulness, and ultimately benefit cognitive function (Herzog et al., 1998); (b) participating in cognitively-focused social activities such as playing mahjong, chess or cards is highly intellectually demanding (Cheng et al., 2006), involving the coordination and of a variety of cognitive abilities, including attention, working memory, reasoning, and calculation (Tyndall et al., 2018). These abilities are repeatedly trained during gameplay, which may maintain or even improve the cognitive function of older adults (Tyndall et al., 2018); (c) Participating in physically focused social activities, such as playing sports, may be associated with a higher level of neurogenesis, such as peripheral vascular endothelial growth factor in the hippocampus (Rich et al., 2017). It may also be associated with lower levels of neuro-immune markers such as C-reactive protein, fibrinogen, and white blood cells that are involved in the cognitive aging process (Tyndall et al., 2018; Walker et al., 2019). Additionally, participating in physical social activities may be linked to lower levels of comorbidity (Atkinson et al., 2023). Due to the trait-like effect of social participation on cognitive function, practitioners are advised to develop interventions that help older adults form a habit of regular social participation to maximize its benefits.

Our research also supports an asymmetrical pattern of time-averaged partner effects: husbands’ social participation was positively associated with their wives’ cognitive function on average (i.e., the partner effect), but wives’ social participation did not significantly predict their husbands’ cognitive function (H2, time-averaged partner effect). Moreover, an increase in neither husbands’ nor wives’ social participation on a particular wave predicted their partner’s cognitive function on that wave (H4, time-specific partner effect), which implies that in terms of the husbands’ partner effect, their social participation may exert a robust influence on their wives’ cognitive function over time without obvious fluctuations. Our asymmetrical pattern is different from the findings of other longitudinal studies addressing a similar question (Hoppmann et al., 2008; Howrey et al., 2021). Howrey et al. (2021) reported that wives’ social participation was associated with husbands’ cognitive function but not vice versa in Mexico, whereas there were no partner effects among American couples. In the United States, couples may prioritize independence over interdependence compared to couples in Mexico, where collectivism is emphasized (Hofstede, 2001). Hoppmann et al. (2008) adopted a latent growth model and found that levels of activities and perceptual speed were positively related, as were husbands’ activity levels and wives’ level of perceptual speed. Thus, there are three distinct patterns of partner effect across Chinese, American, Mexican, and Australian samples: (a) no partner effects in the American sample, (b) asymmetrical partner effect of wives’ social participation on husbands’ cognition in the Mexican sample, and (c) asymmetrical partner effect of husbands’ social participation on wives’ cognition in the Chinese or Australian sample. Contrary to our expectation, we did not find a similar asymmetrical pattern for Chinese and Mexican sample due to similar collectivism social background. These findings suggested that other demographic factors potentially act as moderators or mediators in relation to this link. To illustrate, within the framework of our research, we observed that husbands possessed a higher degree of social privilege in comparison to their wives due to being younger, having a higher level of education, and reporting better health conditions. These factors could potentially reinforce the traditional gendered role and power imbalance within couples. Wives in China may exhibit a higher level of dependency on their husbands in various aspects of their lives, encompassing financial support, decision-making abilities, and social roles and responsibilities. Moreover, this dependency may also extend into cognitive activities. Future studies should examine potential mediators or moderators of the nonreciprocity between members of couples to understand gender differences in response to this research question.

Furthermore, inconsistencies existed in the statistical procedures employed across the studies. Hoppmann et al. (2008) adopted latent growth models to assess the effects of both partners’ cognitive function on their trajectories of social engagement (i.e., actor effects) and each other’s trajectories (i.e., partner effect). In contrast, Howrey et al. (2021) adopted two waves of a large cohort to assess both partners’ baseline social engagement as a predictor of their cognitive function (i.e., actor effect) and each other’s cognitive function (i.e., partner effect) at the follow-up. Our study incorporated all four waves of data via LD-APIM and distinguished between time-average effects and time-specific effects in our analysis. In future research, we recommend assessing this research question using cross-cultural data and consistent analytical protocols.

Our research provides further evidence to support concordance in cognitive function, as demonstrated by significant autoregression estimates (H5), cross-lagged correlations (H6), time-averaged cognitive function between dyads (H7), and wave fluctuation within couples (H8). These findings are consistent with those of other longitudinal studies (Gerstorf et al., 2009; Gruber-Baldini et al., 1995) that suggest husbands and wives share living experiences, environments, and communications, forming interrelated cognition (Ross et al., 2008). Considering their interdependence, psychologists and clinical practitioners should view husbands and wives as a unit (in terms of shared environment and collaboration) when designing couple-based cognition interventions, as it may enhance intervention effects.

Several limitations of the current study should be addressed. First, the baseline characteristics of husbands and wives are not comparable. Although the results remained stable after adjusting for these variables, we cannot eliminate all possible confounders. The observed effects were likely driven by wives’ socioeconomic disadvantages, such as lower education, rural Hukou, and poorer baseline health status. Moreover, our findings may be largely driven by participants with rural Hukou. The over-representation of participants with rural Hukou may limit the extent to which the findings can be generalized to the broader population (e.g., older adults with urban Hukou) since traditional gendered roles are more prevalent in rural areas (Hu & Scott, 2016). Thus, future studies are needed to determine the universality of actor–partner effects among older adults with urban Hukou and the broader population.

Second, we could only create an overall score for social participation due to the uneven distribution of participants’ choices. As most participants chose “interacted with friends,” “played mahjong, chess, or cards or went to a community club,” and “went to a sport, social, or other kind of club,” our findings may primarily reflect the link between these social activities and cognitive function. Moreover, social participation can be evaluated based on various aspects, such as difficulty, independence, frequency, and duration. The most common aspects of social participation assessment include whether the person participated, the degree of limitation, restriction, or difficulty, the degree of independence or need for assistance, and the count of frequency or hours spent on each type of social participation (Hashidate et al., 2021). However, the current study only covers whether the person participated or not and the frequency of participation. Moreover, the rating points for the frequency question regarding frequency may not have been accurate as there were only three choices (“almost daily,” “almost every week,” and “not regularly”). Furthermore, subjective measures such as personal importance and satisfaction can supplement objective measures of social participation.

Third, this study measured limited domains of cognitive function, which may restrict the ability to capture overall cognition. Future studies should measure more cognitive domains, particularly complex executive functions (e.g., problem-solving and reasoning). Moreover, the cognitive function measure (TICS) showed moderate internal reliability at each wave but good test–retest reliability across waves. Future studies use more common cognitive function measures with high internal reliability (e.g., MoCA and MMSE) in China, which may also facilitate result comparison with other studies.

Fourth, this national dataset did not provide information on the extent to which husbands or wives participate in each other’s social activities, making it impossible to test our interpretation of the partner effect. Future studies should customize their surveys to assess this possibility and provide a comprehensive understanding of dyadic interdependence on this topic. Finally, this dataset measured social activities that both partners could participate in, but we were unable to distinguish whether these activities were performed jointly or individually. Howrey et al. (2021) did not separate joint and individual social activities but still found a significant partner effect whereby wives’ social participation benefited their own and their husbands’ cognition only in Mexico, a collectivist culture. These results suggest that people from a collectivist society may be more likely to involve their partners in social activities, which may contribute to partner effects. We acknowledge that the type of participation (joint or individual) may act as both a sign of cultural differences and a moderator that affects the observed effects. Therefore, it is necessary to distinguish the nature of the activity to accurately understand the interrelationships between social participation and cognitive function in the couple.

There are multiple practical implications of the study. First, considering that asymmetrical interdependence exists in older couples, clinical practitioners should encourage both partners to participate in social activities to facilitate crossover benefits and enhance training effects. For example, Mahjong is a game of strategy and skill, usually played with four people, which incorporates multiple cognitive dimensions including attention, memory, and calculation in conjunction with interpersonal social communication (Chu-Man et al., 2015). Moreover, according to this dataset, playing Mahjong is one of the most popular social activities in China. Thus, clinical practitioners may consider Mahjong as an ideal form of complementary alternative medicine and, to enhance its benefits, should invite both dyad members (especially the husband) to participate. Second, policymakers should build more facilities (e.g., community clubs) to encourage older couples to engage in social activities. Encouraging older adults to engage in social activities may be a cost-effective public health policy to buffer cognitive decline and lower the odds of dementia.

Our findings showed an interdependent but asymmetrical pattern of actor–partner interdependence in older Chinese couples, in which husbands’ time-averaged social participation can generally predict their own and their wives’ cognitive function but wives’ time-averaged social participation can only predict their own cognitive function. On the other hand, husbands’ and wives’ average cognitive function and wave fluctuations were highly interrelated. These findings have important implications for both clinical practice and public policy.

## Supplementary Material

gbae045_suppl_Supplementary_Tables_S1-S6_Figures_S1-S3

## Data Availability

This study is not preregistered. This analysis uses data or information from the Harmonized CHARLS dataset and Codebook, Version D as of June 2021 developed by the Gateway to Global Aging Data. The development of the Harmonized CHARLS was funded by the National Institute on Aging (R01 AG030153, RC2AG036619, R03 AG043052). For more information, please refer to https://g2aging.org/. CHARLS is supported by Peking University, the National Natural Science Foundation of China, the National Institute on Aging, and the World Bank. The analytical methods and materials would be available upon reasonable request.
