# Supplementary material for: The Longitudinal Dyadic Associations Between Social Participation and Cognitive Function in Older Chinese Couples
Source: J Gerontol B Psychol Sci Soc Sci. 2024 Apr 12;79(6):gbae045. doi: 10.1093/geronb/gbae045 (PMC11067812; doi:10.1093/geronb/gbae045)
Supplement: gbae045_suppl_Supplementary_Tables_S1-S6_Figures_S1-S3 [file gbae045_suppl_supplementary_tables_s1-s6_figures_s1-s3.docx]

*The Journals of Gerontology, Series B: Psychological Sciences and Social Sciences*, Jianhua Hou, Tianyong Chen, Nancy Xiaonan Yu. The Longitudinal Dyadic Associations between Social Participation and Cognitive Function in Older Chinese Couples**.**

**Figure S1**

*The Screening Process of Included Participants*

**
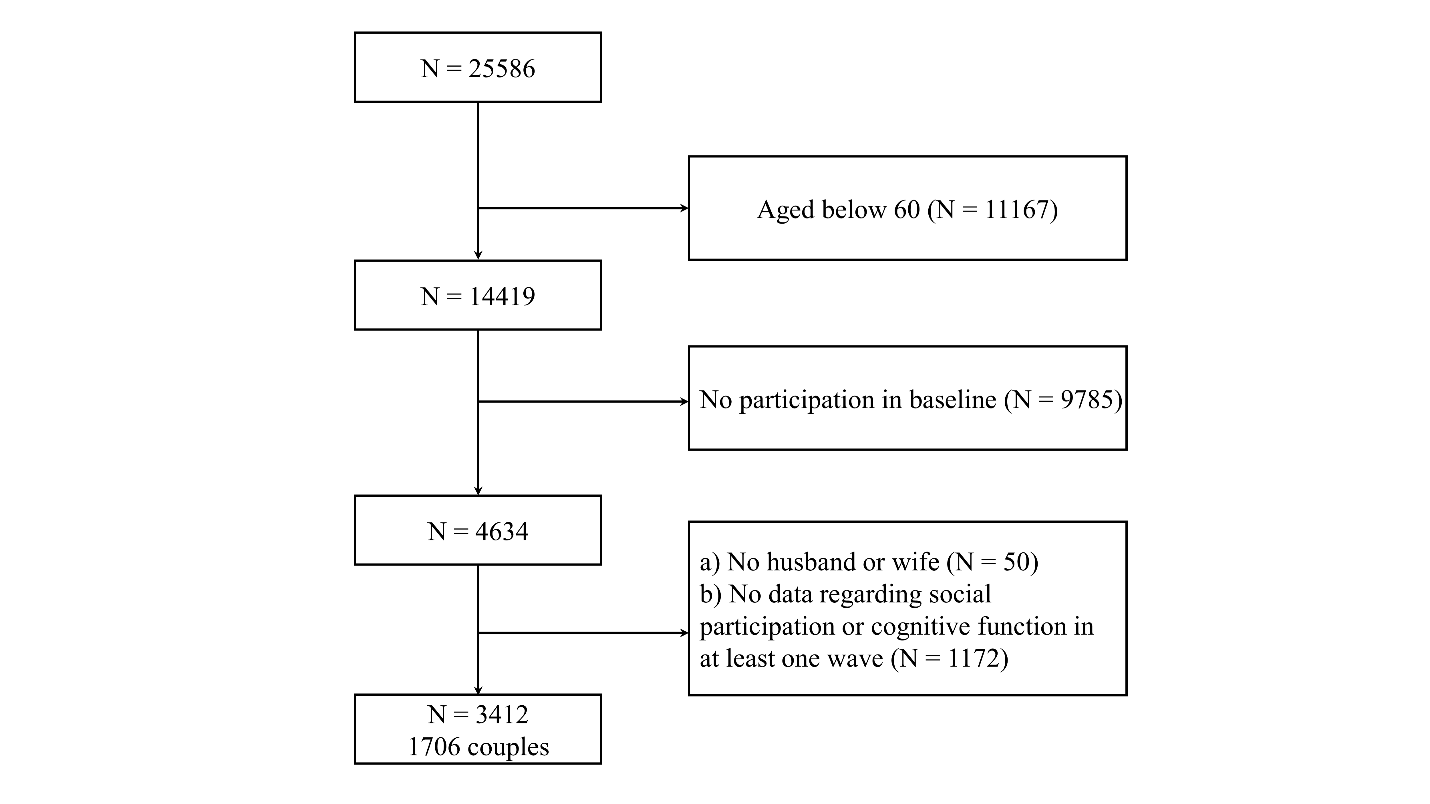
**

**Figure S2**

*The Proportion of Participants Who Participated in Any of the Seven Social Activities*


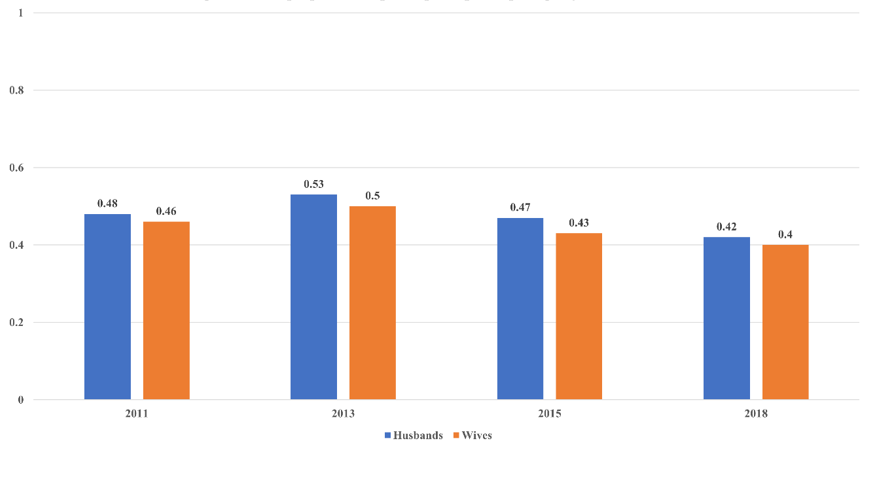


Note:

Seven social activities include a) interacting with friends, b) playing ma-jong, chess, cards or going to community club, c) going to a sport, social, or other kind of club, d) taking part in a community-related organization, e) doing voluntary or charity work, f) caring for a sick or disabled adult who does not live with you and who did not pay you for the help and g) attending an educational or training course.

**Figure S3**

*The Distribution of Social Activities*


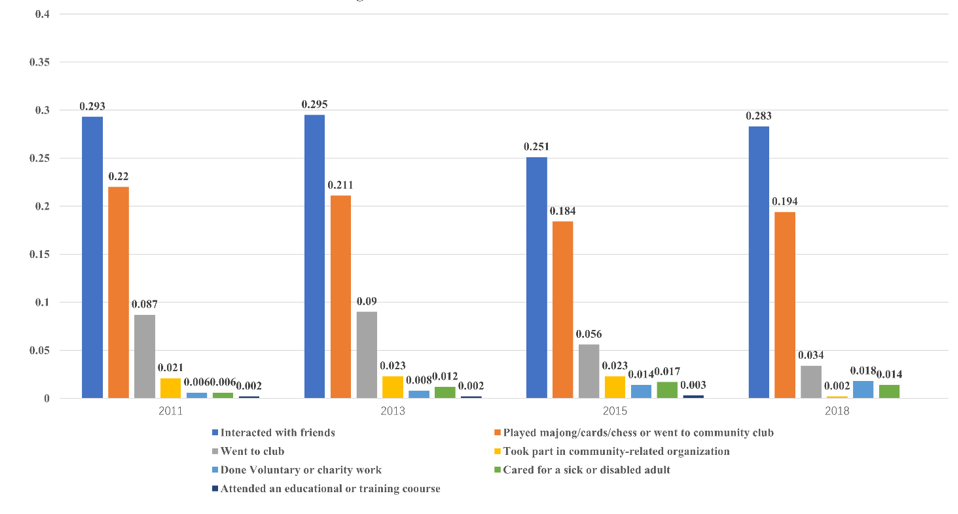


**Table S1**

*STROBE Checklist for the Cohort Study*

|  | Item No | Recommendation | Page No |
| --- | --- | --- | --- |
| **Title and abstract** | 1 | (*a*) Indicate the study’s design with a commonly used term in the title or the abstract | 1 |
|  |  | (*b*) Provide in the abstract an informative and balanced summary of what was done and what was found | 2 |
| Introduction | | | |
| Background/rationale | 2 | Explain the scientific background and rationale for the investigation being reported | 5-6 |
| Objectives | 3 | State specific objectives, including any prespecified hypotheses | 7 |
| Methods | | | |
| Study design | 4 | Present key elements of study design early in the paper | 8 |
| Setting | 5 | Describe the setting, locations, and relevant dates, including periods of recruitment, exposure, follow-up, and data collection | 8 |
| Participants | 6 | (*a*) Give the eligibility criteria, and the sources and methods of selection of participants. Describe methods of follow-up | 8 |
|  |  | (*b*) For matched studies, give matching criteria and number of exposed and unexposed | 8 |
| Variables | 7 | Clearly define all outcomes, exposures, predictors, potential confounders, and effect modifiers. Give diagnostic criteria, if applicable | 8-9 |
| Data sources/ measurement | 8* | For each variable of interest, give sources of data and details of methods of assessment (measurement). Describe comparability of assessment methods if there is more than one group | 8-9 |
| Bias | 9 | Describe any efforts to address potential sources of bias | 8 |
| Study size | 10 | Explain how the study size was arrived at | 8 |
| Quantitative variables | 11 | Explain how quantitative variables were handled in the analyses. If applicable, describe which groupings were chosen and why | 8-9 |
| Statistical methods | 12 | (*a*) Describe all statistical methods, including those used to control for confounding | 10 |
|  |  | (*b*) Describe any methods used to examine subgroups and interactions | NA |
|  |  | (*c*) Explain how missing data were addressed | 10 |
|  |  | (*d*) If applicable, explain how loss to follow-up was addressed | 10 |
|  |  | (*e*) Describe any sensitivity analyses | 10 |
| Results | | |  |
| Participants | 13* | (a) Report numbers of individuals at each stage of study—eg numbers potentially eligible, examined for eligibility, confirmed eligible, included in the study, completing follow-up, and analysed | Figure S1 |
|  |  | (b) Give reasons for non-participation at each stage |  |
|  |  | (c) Consider use of a flow diagram |  |
| Descriptive data | 14* | (a) Give characteristics of study participants (e.g., demographic, clinical, social) and information on exposures and potential confounders | 11 |
|  |  | (b) Indicate number of participants with missing data for each variable of interest |  |
|  |  | (c) Summarise follow-up time (e.g., average and total amount) |  |
| Outcome data | 15* | Report numbers of outcome events or summary measures over time | 11 |

**Table S2**

*The Distribution of Social Participation Across Four Waves*

|  | Wave 1 | Wave 2 | Wave 3 | Wave 4 |
| --- | --- | --- | --- | --- |
| Husbands |  |  |  |  |
| Not attending social activities | 854 (52.2%) | 673 (46.3%) | 738 (53.4%) | 716 (57.0%) |
| Attending any social activities almost once every week or not regularly | 489 (29.9%) | 457 (31.4%) | 411 (29.7%) | 334 (26.6%) |
| Attending any social activities almost daily | 293 (17.9%) | 325 (22.3%) | 234 (16.9%) | 206 (16.4%) |
| Wives |  |  |  |  |
| Not attending social activities | 899 (54.4%) | 743 (49.6%) | 804 (55.7%) | 817 (58.1%) |
| Attending any social activities almost once every week or not regularly | 419 (25.4%) | 391 (26.1%) | 376 (26.0%) | 332 (23.6%) |
| Attending any social activities almost daily | 334 (20.2%) | 363 (24.2%) | 264 (18.3%) | 257 (18.3%) |

**Table S3**

*Parameter Estimates of the Fixed Effects Adjusted by Age, Education, Hukou, Self-reported Health status, and Depression*

|  | *b* | *SE* | *p* | *Lower CI* | *Upper CI* |
| --- | --- | --- | --- | --- | --- |
| **Husbands’ cognitive function** |  |  |  |  |  |
| Intercept | **11.28** | 0.84 | < .001 | 9.63 | 12.93 |
| Lagged | **0.13** | 0.04 | < .001 | 0.06 | 0.19 |
| Cross-lagged | **0.11** | 0.03 | .001 | 0.04 | 0.17 |
| Husbands’ social participation_ time averaged | **0.74** | 0.17 | < .001 | 0.41 | 1.08 |
| Wives’ social participation_ time averaged | 0.06 | 0.16 | .718 | −0.26 | 0.38 |
| Husbands’ social participation_ time specific | 0.17 | 0.11 | .128 | −0.05 | 0.39 |
| Wives’ social participation_ time specific | −0.03 | 0.11 | .795 | −0.25 | 0.19 |
| **Wives’ cognitive function** |  |  |  |  |  |
| Intercept | **9.93** | 0.94 | < .001 | 8.09 | 11.78 |
| Lagged | **0.19** | 0.04 | < .001 | 0.11 | 0.28 |
| Cross-lagged | 0.02 | 0.04 | .669 | −0.06 | 0.09 |
| Wives’ social participation_ time averaged | **0.78** | 0.19 | < .001 | 0.41 | 1.15 |
| Husbands’ social participation_ time averaged | **0.49** | 0.19 | .011 | 0.11 | 0.86 |
| Wives’ social participation_ time specific | 0.05 | 0.13 | .717 | −0.20 | 0.29 |
| Husbands’ social participation_ time specific | 0.17 | 0.14 | .231 | −0.11 | 0.44 |

**Table S4**

*Parameter Estimates of the Covariance Structure Adjusted by Age, Education, Hukou, Self-reported Health Status, and Depression*

|  | *ϕ* | *SE* | *p* | *Lower CI* | *Upper CI* |
| --- | --- | --- | --- | --- | --- |
| Between-dyad covariance |  |  |  |  |  |
| $\tau_{h}^{2}$ | 5.51 | 0.78 | **< .001** | 3.97 | 7.04 |
| $\tau_{w}^{2}$ | 7.65 | 1.22 | **< .001** | 5.26 | 10.03 |
| $\tau_{hw}$ | 0.84 | 0.78 | .283 | −0.69 | 2.37 |
| Within-dyad covariance |  |  |  |  |  |
| $\sigma_{h}^{2}$ | 10.94 | 0.45 | **< .001** | 10.06 | 11.82 |
| $\sigma_{w}^{2}$ | 12.53 | 0.58 | **< .001** | 11.40 | 13.67 |
| $\sigma_{hw}$ | 1.96 | 0.42 | **< .001** | 1.13 | 2.79 |

**Table S5**

*Parameter estimates of the fixed effects using a dataset without death-caused missing*

|  | *b* | *SE* | *p* | *Lower CI* | *Upper CI* |
| --- | --- | --- | --- | --- | --- |
| **Husbands’ cognitive function** |  |  |  |  |  |
| Intercept | **9.22** | 0.61 | < .001 | 8.02 | 10.42 |
| Lagged | **0.16** | 0.04 | < .001 | 0.09 | 0.24 |
| Cross-lagged | **0.10** | 0.04 | .004 | 0.03 | 0.17 |
| Husbands’ social participation_ time averaged | **1.13** | 0.19 | < .001 | 0.76 | 1.49 |
| Wives’ social participation_ time averaged | 0.15 | 0.18 | .394 | −0.20 | 0.50 |
| Husbands’ social participation_ time specific | 0.18 | 0.12 | .123 | −0.05 | 0.41 |
| Wives’ social participation_ time specific | −0.04 | 0.12 | .714 | −0.27 | 0.19 |
| **Wives’ cognitive function** |  |  |  |  |  |
| Intercept | **8.67** | 0.68 | < .001 | 7.34 | 9.99 |
| Lagged | **0.17** | 0.04 | < .001 | 0.08 | 0.26 |
| Cross-lagged | 0.01 | 0.04 | .830 | −0.07 | 0.08 |
| Husbands’ social participation_ time averaged | **1.03** | 0.21 | < .001 | 0.61 | 1.44 |
| Wives’ social participation_ time averaged | **0.76** | 0.22 | < .001 | 0.33 | 1.19 |
| Husbands’ social participation_ time specific | 0.03 | 0.13 | .826 | −0.22 | 0.28 |
| Wives’ social participation_ time specific | 0.19 | 0.14 | .176 | −0.08 | 0.46 |

**Table S6**

*Parameter Estimates of the Covariance Structure Using a Dataset Without Death-caused Missing*

|  | *ϕ* | *SE* | *p* | *Lower CI* | *Upper CI* |
| --- | --- | --- | --- | --- | --- |
| Between-dyad covariance |  |  |  |  |  |
| $\tau_{h}^{2}$ | 6.77 | 1.05 | **< .001** | 4.72 | 8.82 |
| $\tau_{w}^{2}$ | 10.71 | 1.58 | **< .001** | 7.62 | 13.80 |
| $\tau_{hw}$ | 2.82 | 1.00 | **.005** | 0.86 | 4.79 |
| Within-dyad covariance |  |  |  |  |  |
| $\sigma_{h}^{2}$ | 11.42 | 0.50 | **< .001** | 10.49 | 12.45 |
| $\sigma_{w}^{2}$ | 12.47 | 0.59 | **< .001** | 11.32 | 13.63 |
| $\sigma_{hw}$ | 2.02 | 0.46 | **< .001** | 1.15 | 2.89 |
